# Supplementary material for: Large piezoelectric response in a Jahn-Teller distorted molecular metal halide
Source: Nat Commun. 2023 Apr 3;14:1852. doi: 10.1038/s41467-023-37471-3 (PMC10070272; doi:10.1038/s41467-023-37471-3)
Supplement: Supplementary file 1 — Supplementary Information [file 41467_2023_37471_MOESM1_ESM.pdf]

Supplemental Information:

Large piezoelectric response in a Jahn-Teller distorted molecular metal halide

Sasa Wang[1,†], Asif Abdullah Khan[2,3,†], Sam Teale[1,†], Jian Xu[1,†], Darshan H. Parmar[1], Ruyan Zhao[4], Luke Grater[1] Peter Serles[5], Yu Zou[6], Tobin Filleter[5], Dwight S. Seferos[4], Dayan Ban[2,3], and Edward H. Sargent[1*]*

[1]*Department of Electrical and Computer Engineering, University of Toronto, 10 King's College Road, Toronto, Ontario M5S 3G4, Canada.*

[2]*Waterloo Institute for Nanotechnology, University of Waterloo, 200 University Ave West, Waterloo, Ontario N2L 3G1, Canada.*

[3]*Department of Electrical and Computer Engineering, University of Waterloo, 200 University Ave West, Waterloo, Ontario N2L 3G1, Canada.*

[4]*Department of Chemistry, University of Toronto, 80 St. George Street, Toronto, Ontario M5S 3H6, Canada.*

[5]*Department of Mechanical & Industrial Engineering, University of Toronto, 5 King's College Road, Toronto, Ontario M5S 3G8, Canada.*

[6]*Department of Materials Science and Engineering, University of Toronto, 184 College Street, Toronto, Ontario M5S 3E4, Canada.*

[†]*These authors contributed equally.*

[*]*Correspondence to: Edward H. Sargent (ted.sargent@utoronto.ca), Dayan Ban (dban@uwaterloo.ca)*

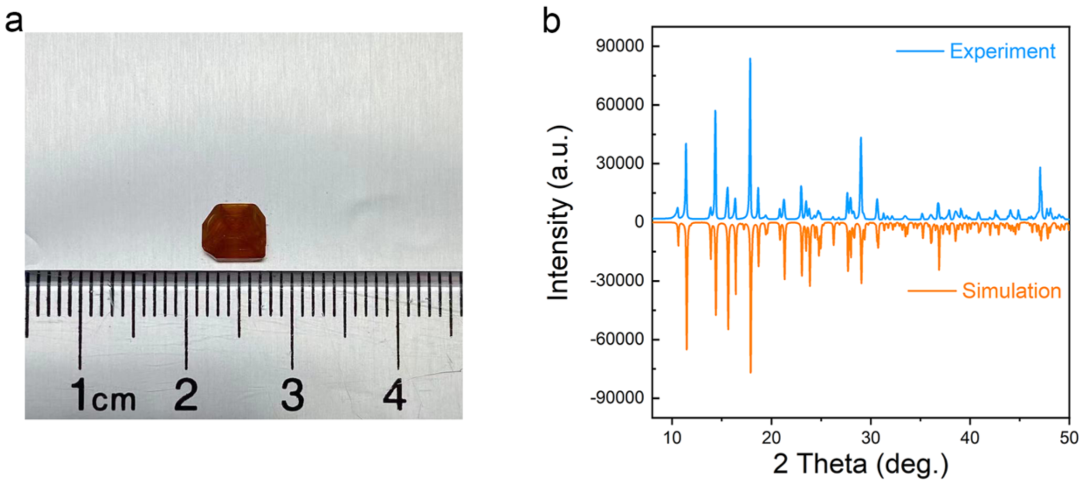

Figure S1. Single Crystal morphology and XRD patterns of EDABCO-$CuCl_4$. (a) Bulk single crystal of EDABCO-$CuCl_4$ grown by the slow-cooling method. (b) Experiment and simulated powder XRD patterns of EDABCO-$CuCl_4$ at room temperature.

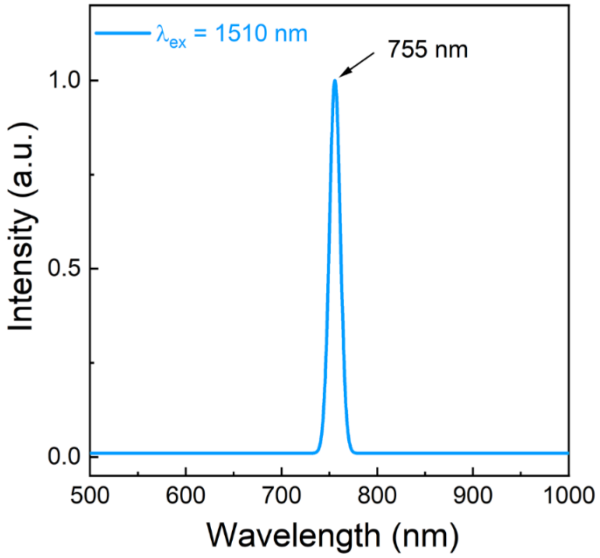

Figure S2. Determination of non-centrosymmetric property of EDABCO-$CuCl_4$. SHG signal of EDABCO-$CuCl_4$ microcrystals at an excitation wavelength of 1510 nm.

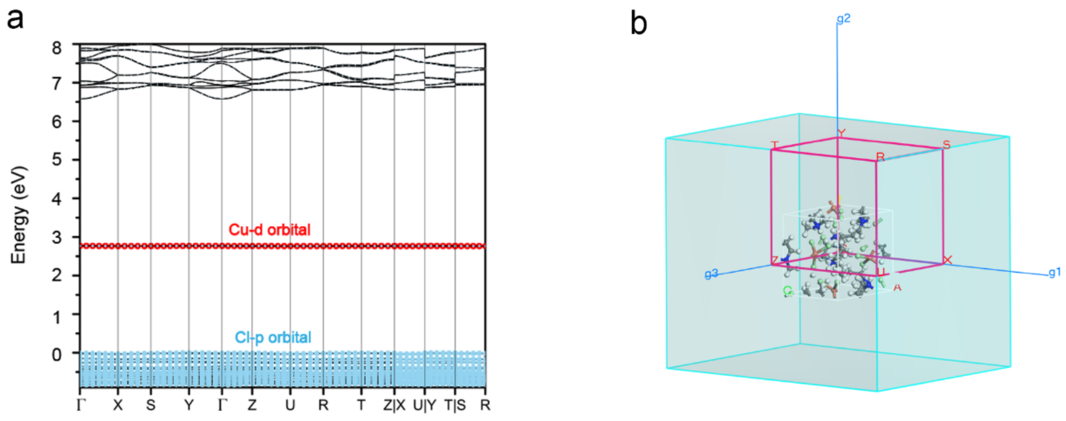

Figure S3. DFT calculated band structure of EDABCO-$CuCl_4$. (a) Calculated band structure and (b) the Brillouin zone of EDABCO-$CuCl_4$.

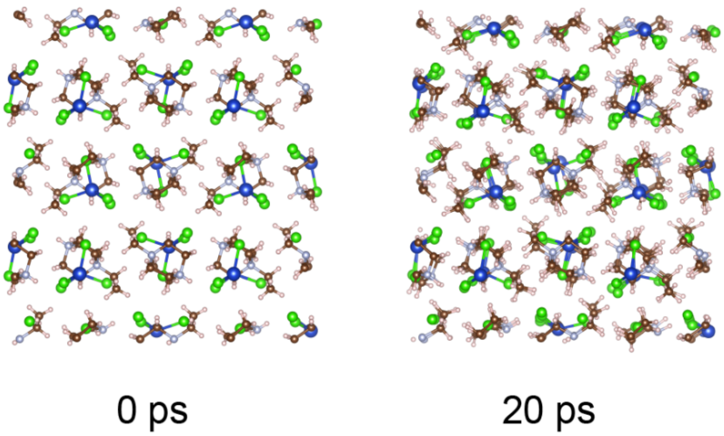

Figure S4. AIMD simulated average structure of EDABCO-$CuCl_4$. Snapshots of the EDABCO-$CuCl_4$ structure at 0 ps (initial frame) and 20 ps (final frame) at 453 K from AIMD simulations.

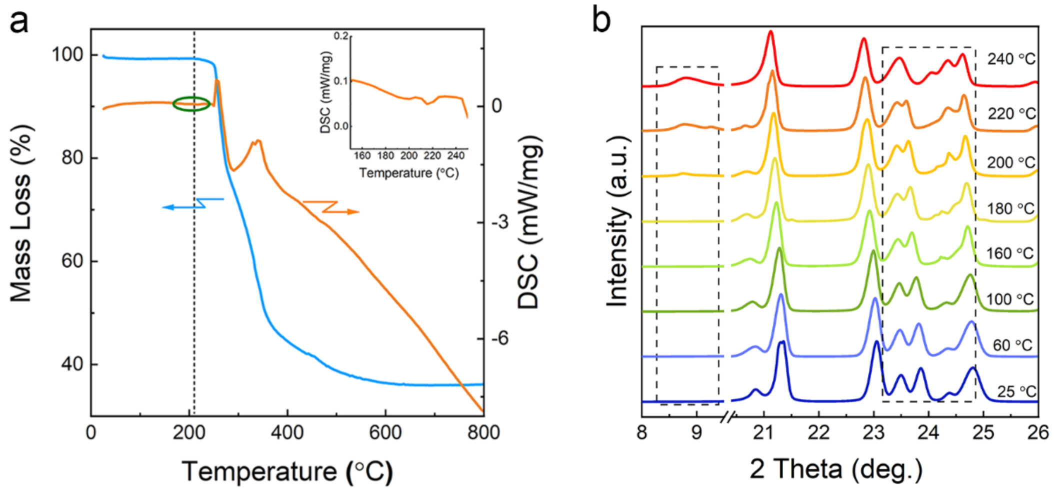

Figure S5. Thermal properties of EDABCO-$CuCl_4$. (a) TG-DSC curves of EDABCO-$CuCl_4$; inset: the zoom-in DSC curve in the temperature range of 150-250 ºC. (b) Temperature-dependent powder XRD patterns of EDABCO-$CuCl_4$. The DSC curve shows that the melting process occurs at 200-226 ºC, which is accompanied by a weight loss at 210 °C as revealed by the TG curve. The temperature-dependent powder XRD patterns indicate no solid-state structural phase transition before its melting point.

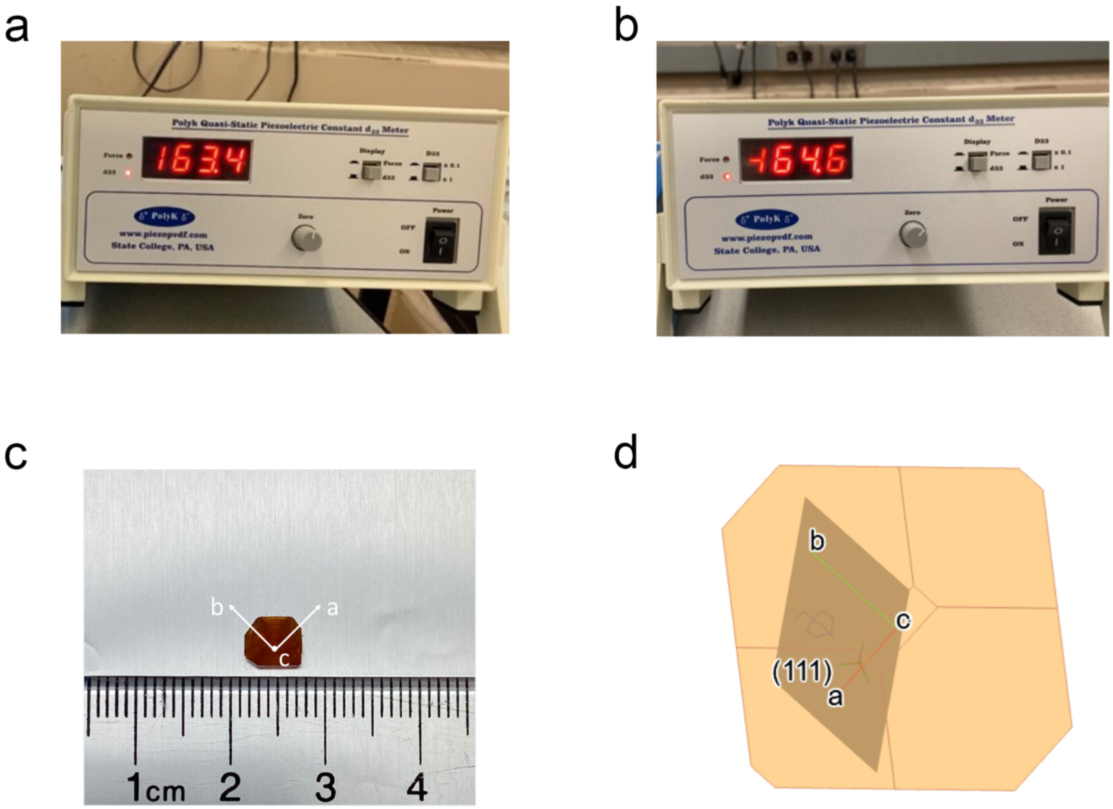

Figure S6. Piezoelectric response and orientation of EDABCO-$CuCl_4$ crystal. (a, b) Photos of the d_{33} measurement on the single crystal using a d_{33} meter, which shows the maximum d_{33} of EDABCO-$CuCl_4$. (c) Bulk single crystal of EDABCO-$CuCl_4$, the largest natural face is the (001) direction. (d) Schematic of the (111) direction in the crystal. The morphology is calculated according to the single-crystal structure data.

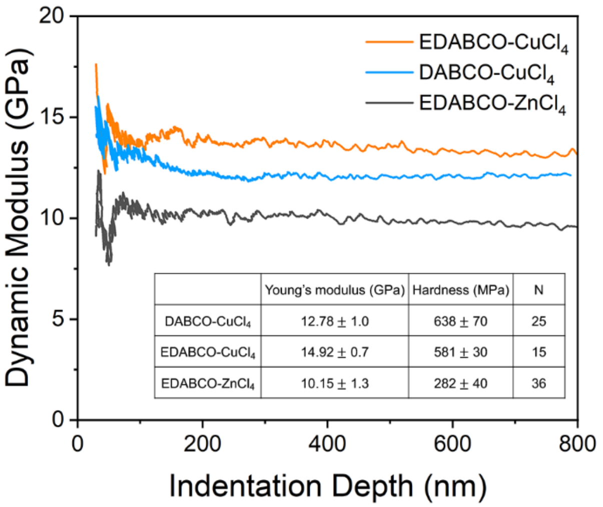

Figure S7. Elastic properties of DABCO-$CuCl_4$, EDABCO-$CuCl_4$, and EDABCO-$ZnCl_4$. Young's modulus and hardness of DABCO-$CuCl_4$, EDABCO-$CuCl_4$, and EDABCO-$ZnCl_4$ single crystals. The Young's modulus and hardness are significantly smaller than typical ceramic piezoelectric materials such as $BaTiO_3$ (E = 100 ~130 GPa, H = 4~5 GPa) and PZT (E = 60 ~90 GPa, H = 4~7 GPa).

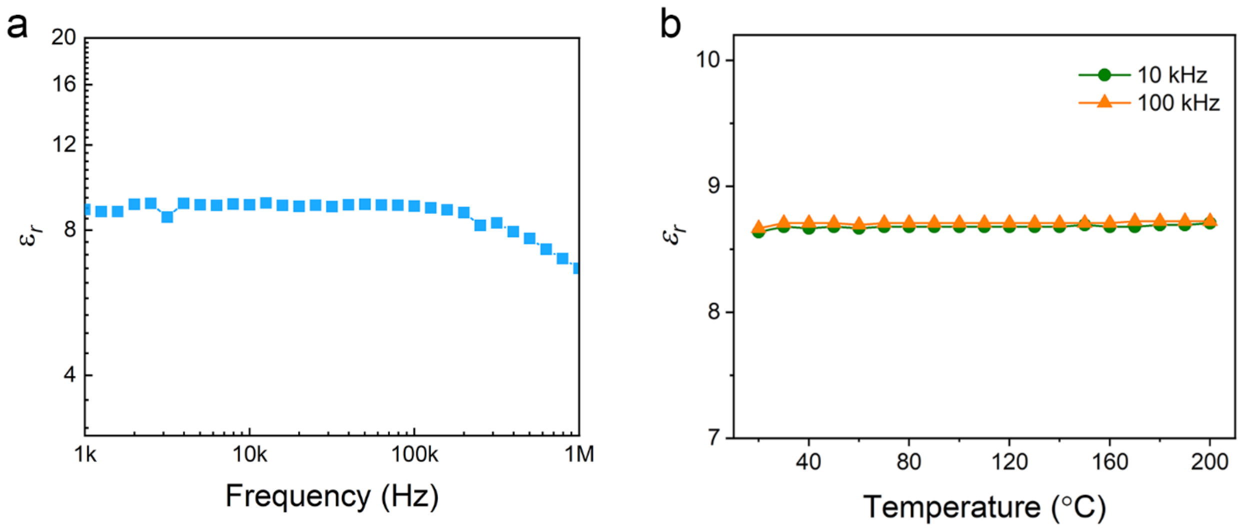

Figure S8. Dielectric properties of EDABCO-$CuCl_4$. (a) Dielectric constant as a function of the frequency of EDABCO-$CuCl_4$ single crystals. (b) Temperature-dependent data of the dielectric constant at different frequencies.

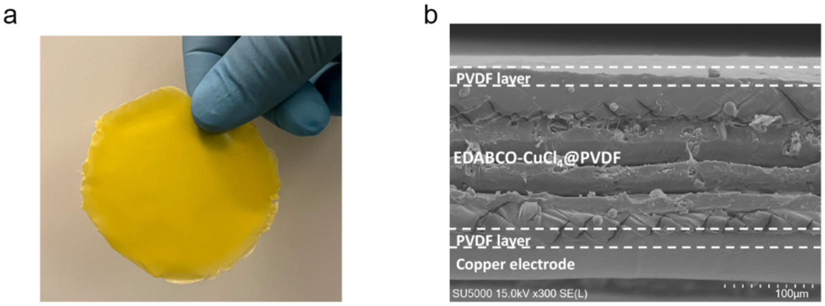

Figure S9. Images and composite of the nanogenerator device. (a) Picture and (b) the cross-sectional SEM image of a 10 wt.% EDABCO-$CuCl_4$@PVDF composite film.

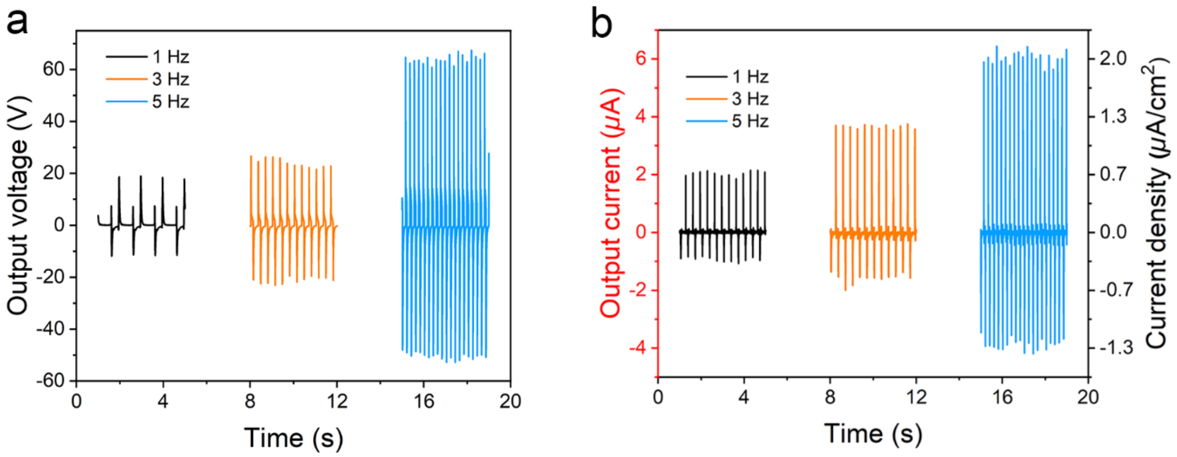

Figure S10. Frequency-dependent energy harvesting properties. (a) The output voltage and (b) the output current of the 10 wt.% EDABCO-$CuCl_4$@PVDF piezoelectric generators with different frequencies (1 Hz, 3 Hz, and 5 Hz) at 15 N.

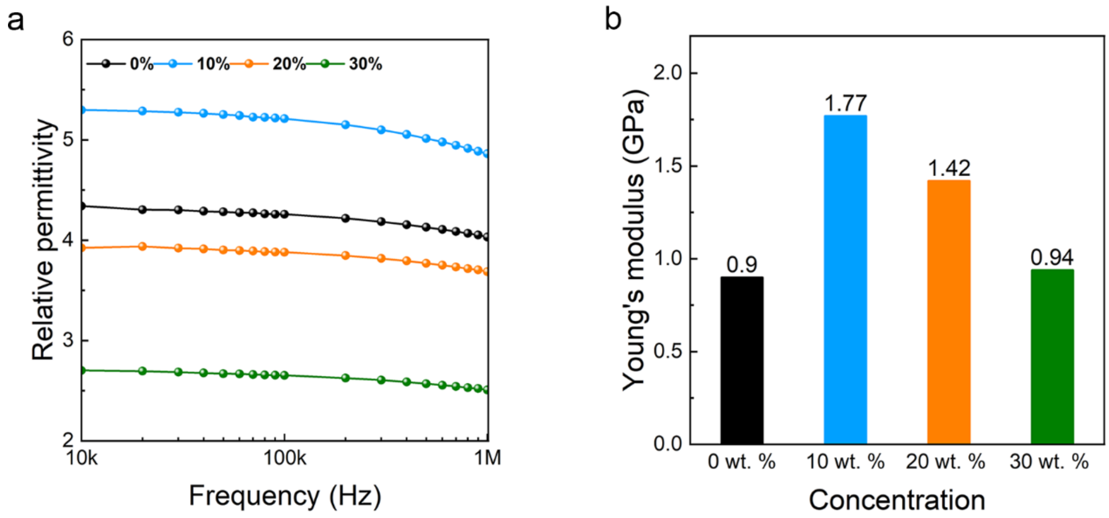

Figure S11. Variation of the dielectric and elastic properties with EDABCO-$CuCl_4$ concentration. (a) The relative permittivity of PVDF and EDABCO-$CuCl_4$@PVDF composite films as a function of frequency. The decrease in the permittivity at higher EDABCO-$CuCl_4$ concentrations is attributed to particle aggregation that leads to the randomization of dipoles. (b) Young's modulus of PVDF and EDABCO-$CuCl_4$@PVDF composite films (10 wt.%, 20 wt.%, 30 wt.%). A greater Young's modulus is desirable to enhance the domain mobility of the localized stress points, thus contributing to the increase in overall polarization.

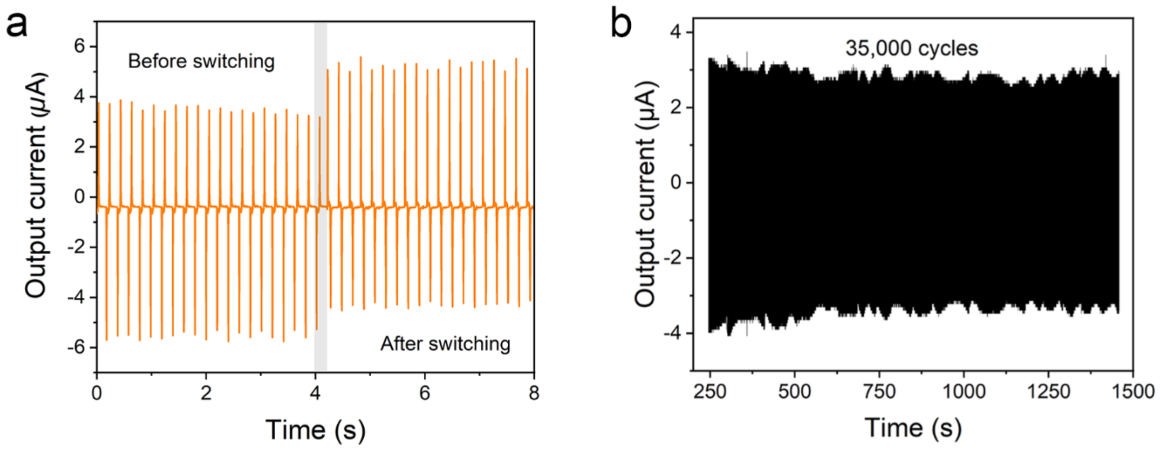

Figure S12. Polarity switching and reliability properties of the PG. (a) Polarity switching of the output current of the 10 wt.% EDABCO-$CuCl_4$@PVDF PG. (b) The device reliability test for 35,000 cycles of the 10 wt.% EDABCO-$CuCl_4$@PVDF PG at 30 Hz with a 136-gram weight on top of the device.

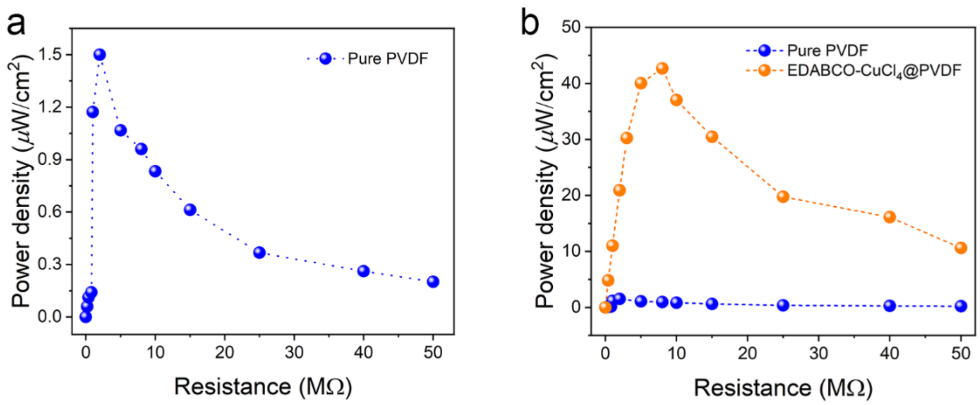

Figure S13. Comparison of the power density between pure PVDF and the PG. (a) The instantaneous power density of a pure PVDF PG with load resistance ranges from 0 to 50 MΩ. (b) Comparison of the power density of a pure PVDF and EDABCO-$CuCl_4$@PVDF PG.

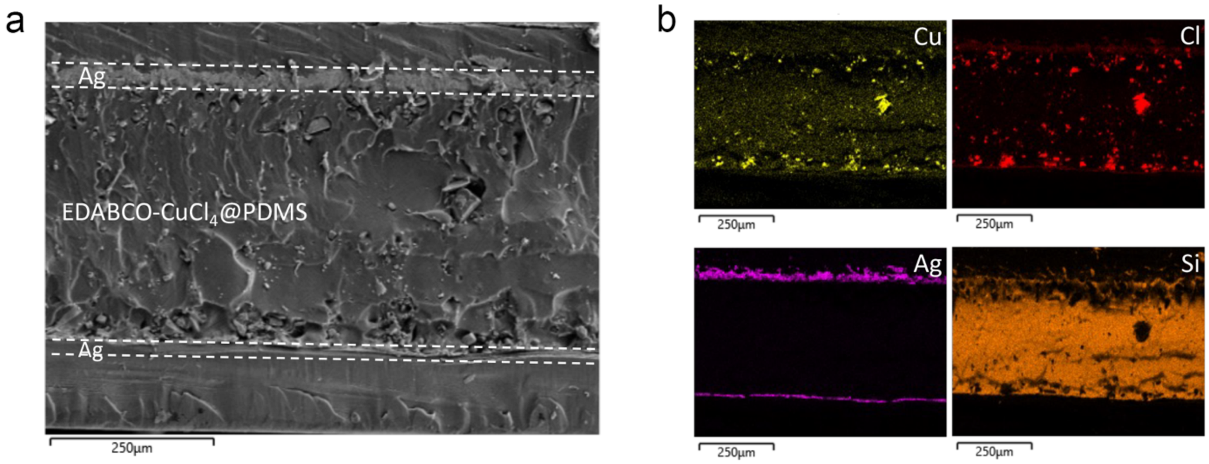

Figure S14. Cross-sectional SEM and EDS mapping of the PG. (a) The cross-sectional SEM and (b) EDS mapping of EDABCO-$CuCl_4$@PDMS piezoelectric generator after operating at 433 K for ~2,500 cycles.

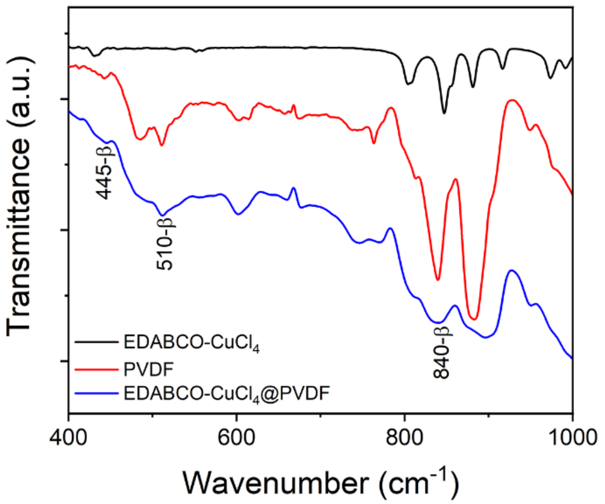

Figure S15. Illustrates the beta (β)-phase of PVDF. FTIR transmission spectra of EDABCO-$CuCl_4$, pure PVDF, and the 10 wt. % EDABCO-$CuCl_4$@PVDF film. The characteristic peaks at 445, 510 and 840 cm^{-1} correspond to the beta (β)-phase of PVDF. The β-phase content for pure PVDF is estimated to be 80% and for the composite film is 85%. Given that pure PVDF generates a V_{oc} of 25 V, we associate the contribution from the increased 5% β-phase PVDF to be roughly 1.5 V, whereas an increase of 38 V is observed for the composite film. Compared to the output voltage of ~ 63 V in the 10 wt. % composite with PVDF, the 5 wt. % EDABCO-$CuCl_4$@PDMS generates an output voltage of ~ 20 V. This is a quantity that could potentially be enhanced by increasing the nanoparticle concentration and controlling their dispersion. We also note that the lower Young's modulus of PDMS (1.4 MPa) than PVDF will consume more mechanical stress and reduce the stress transfer efficiency to the EDABCO-$CuCl_4$ NPs.

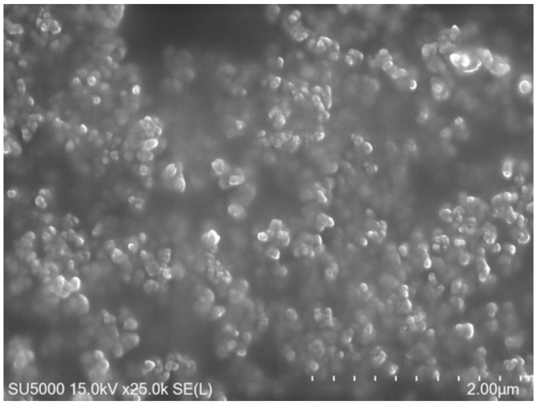

Figure S16. Morphology of the EDABCO-$CuCl_4$ nanoparticles. SEM image of the finely ground EDABCO-$CuCl_4$ nanoparticles.

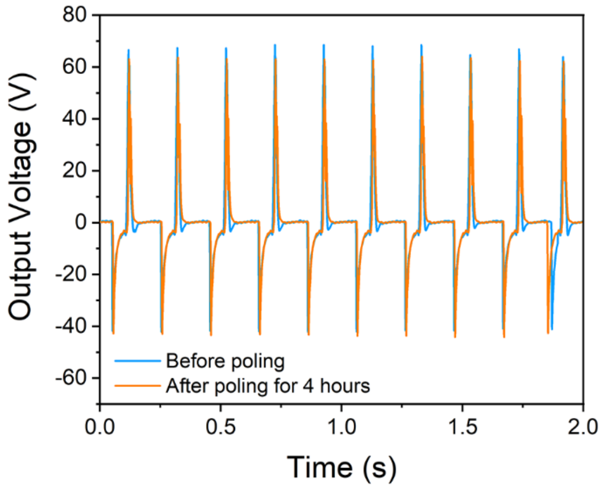

Figure S17. Demonstrating the effect of poling on the output performance. Comparison of the output voltage of the unpoled and poled EDABCO-$CuCl_4$@PVDF film.

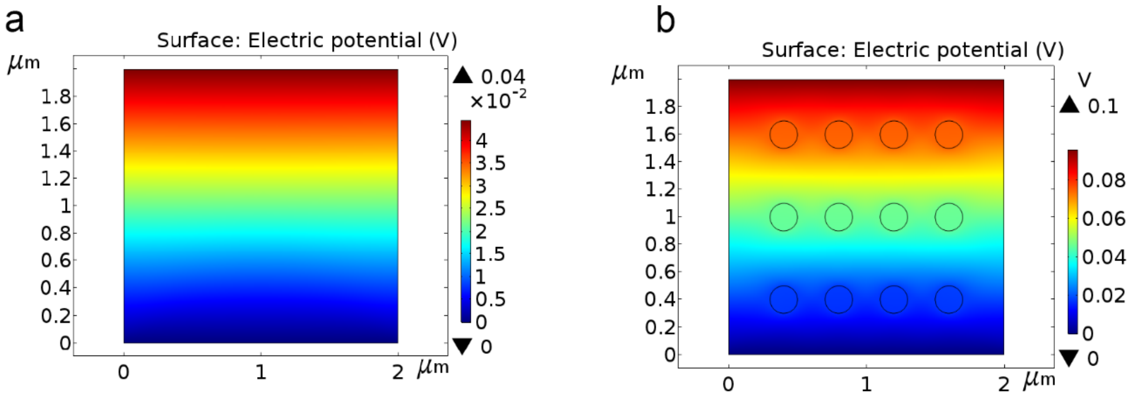

Figure S18. Calculated piezoelectric potential distribution. Piezoelectric potential distribution in (a) pure PVDF and (b) 10% EDABCO-$CuCl_4$@PVDF composite film calculated by COMSOL Multiphysics 5.3. For the pure PVDF film, the calculated piezoelectric potential is 0.04 V at an applied pressure of 50 kPa. After introducing nanoparticles (approximately 10% of PVDF) to form the composite film, the calculated piezoelectric potential changes to 0.1 V, 2.5 times that of the pure PVDF film. This value is consistent with the value seen in the 10% EDABCO-$CuCl_4$@PVDF device, where a ratio of ~2.5 is obtained by comparing the V_{oc} of the composite film and pure PVDF.

Table S1. List of d_{33}, g_{33}, operating temperature limit (T), and energy density of previously reported piezoelectric.

Piezoelectric material	d_{33} (pm/V)	g_{33} (10^{-3} V m N^{-1})	T (°C)	$d_{33}*g_{33}$ (10^{-12} m^3 J^{-1})	Toxicity	Ref.
$BaTiO_3$	125	11	120	1.38	No	1
PZT-4	289	26.1	328	7.54	Yes	2
$PbTiO_3$ single crystal	143	129	-	18.5	Yes	3
Sm,Mn-doped $PbTiO_3$	59	34	321	2	Yes	4
KNN-ceramic	275	30.26	288	8.32	Yes	5
PMN-33%PT	2200	44	145	96.8	Yes	6
SM-PT	127	115	364	14.6	Yes	7
TGS	22	37	49	0.834	No	8
Imidazolium perchlorate (IP)	41	154.4	100.6	6.31	No	9
PVDF	30	286	50	8.58	No	10
DIPAB	11	15.5	153	0.17	No	11
$(ATHP)_2PbBr_4$	75	660	230	49.5	Yes	12
$C_6H_5N(CH_3)_3CdBr_2Cl_{0.75}I_{0.25}$	367	3595	58	1222.8	Yes	13
$(TMCM)_2SnCl_6$	137	980	92	134.26	No	14
**EDABCO-$CuCl_4$**	**165**	**2110**	**180**	**348**	**No**	**This work**

Supplementary Note 1:

As there is no universal performance metric to compare the piezoelectric energy harvesters, the output performance is highly dependent on the operating conditions, dimensions, and materials employed. To emphasize a more logical performance-metric for hybrid metal halides-based composite energy harvesters, in addition to its areal power density we considered the input mechanical energy factors as well (force, and frequency). As the output power will be scaled up with the applied force, and frequency (at a range of low frequency), this will qualitatively reflect a more accurate comparison of different energy harvesters.

Table S2. Piezoelectric energy harvesting based on hybrid metal halide composites.

Hybrid Composites	Power Density (μW/ (cm^2 · N · Hz))	Toxicity	Ref.
$[BnNMe_3]_2CdBr_4$-PDMS	2.3 ×10^{-2}	Yes	[15]
$FAPbBr_3$-PVDF	6.5 ×10^{-2}	Yes	[16]
$(ATHP)_2PbBr_2Cl_2$-PDMS	1×10^{-2}	Yes	[17]
$TMCM_2SnCl_6$-PDMS	1.3 ×10^{-2}	No	[14]
$(Ph_3PMe)_4[CuCl_4]$-TPU	9×10^{-2}	No	[18]
$FASnI_3$-PVDF	4.3 ×10^{-2}	No	[19]
$MASnBr_3$-PDMS	3×10^{-2}	No	[20]
$FAPbBr_2I$-PVDF	8×10^{-2}	Yes	[21]
$MAPbI_3$−PVDF	2.6×10^{-3}	yes	[22]
$FASnBr_3$-PDMS	0.11	no	[23]
$MASnI_3$-PVDF	0.43	No	[24]
$[Ph_3MeP]_4[Ni(NCS)_6]$-TPU	0.37	No	[25]
**EDABCO-$CuCl_4$-PVDF**	**0.57****	**No**	**This work**

** The measured output power density from the PVDF PG was 1.5 μW/cm^2. By discarding the contribution from pure PVDF, the normalized power density of the EDABCO-$CuCl_4$-PVDF PG becomes 0.55.

Table S3. Crystal data and structure refinement for EDABCO-$CuCl_4$.

Empirical formula	C8 H18 Cl4 Cu N2
Formula weight	347.58
Temperature	290(2) K
Wavelength	0.71073 Å
Crystal system	Orthorhombic
Space group	$P2_12_12_1$
Unit cell dimensions	a = 9.8975(11) Å α= 90°<br>b = 11.3273(13) Å β= 90°<br>c = 12.2917(15) Å γ = 90°
Volume	1378.0(3) $Å^3$
Z	4
Density (calculated)	1.675 mg/m^3
Absorption coefficient	2.332 mm^{-1}
F(000)	708
Crystal size	0.280*0.150*0.090 mm^3
Theta range for data collection	2.445 to 27.471°
Index ranges	$-11 \le h \le 12$, $-14 \le k \le 14$, $-15 \le l \le 15$
Reflections collected	24307
Independent reflections	3157 [R(int) = 0.0378]
Completeness to theta = 25.242°	99.4 %
Absorption correction	Semi-empirical from equivalents
Max. and min. transmission	0.7456 and 0.6305
Refinement method	Full-matrix least-squares on F^2
Data / restraints / parameters	3157 / 1 / 140
Goodness-of-fit on F^2	1.092
Final R indices [I>2sigma(I)]	R1 = 0.0249, wR2 = 0.0573
R indices (all data)	R1 = 0.0312, wR2 = 0.0589
Absolute structure parameter	0.005(6)
Largest diff. peak and hole	0.263 and -0.462 e. $Å^{-3}$

Table S4. Hydrogen bonds for EDABCO-$CuCl_4$ [Å and °].

D–H···A	d(D–H)	d(H···A)	d(D···A)	<(DHA)
N(2)–H(2N)···Cl(3)	0.854(13)	2.53(2)	3.247(3)	142(3)
N(2)–H(2N)···Cl(4)	0.854(13)	2.73(3)	3.308(3)	127(3)

Table S5. Cu-Cl bond lengths [Å] and Cl-Cu-Cl angles [°] for EDABCO-$CuCl_4$.

Cu(1)-Cl(1)	2.2175(10)
Cu(1)-Cl(2)	2.2473(9)
Cu(1)-Cl(4)	2.2548(9)
Cu(1)-Cl(3)	2.2568(9)
Cl(1)-Cu(1)-Cl(2)	100.94(4)
Cl(1)-Cu(1)-Cl(4)	132.36(4)
Cl(2)-Cu(1)-Cl(4)	99.15(4)
Cl(1)-Cu(1)-Cl(3)	102.65(4)
Cl(2)-Cu(1)-Cl(3)	130.72(4)
Cl(4)-Cu(1)-Cl(3)	95.87(4)

Table S6. Atomic coordinates ($\times 10^4$) and equivalent isotropic displacement parameters ($Å^2 \times 10^3$) for EDABCO-$CuCl_4$. U(eq) is defined as one-third of the trace of the orthogonalized U_{ij} tensor.

	x	y	z	U(eq)
Cu(1)	4979(1)	395(1)	5807(1)	31(1)
Cl(1)	4555(1)	23(1)	4069(1)	51(1)
Cl(2)	5780(1)	-1362(1)	6360(1)	43(1)
Cl(3)	3130(1)	1413(1)	6331(1)	40(1)
Cl(4)	6488(1)	1585(1)	6635(1)	36(1)
N(1)	4828(3)	5716(2)	4695(2)	28(1)
N(2)	4673(2)	3892(2)	5864(2)	30(1)
C(1)	6093(3)	5583(3)	5374(3)	32(1)
C(2)	5878(3)	4600(3)	6195(3)	36(1)
C(3)	3643(3)	5794(3)	5454(3)	37(1)
C(4)	3418(3)	4596(3)	5988(3)	38(1)
C(5)	4670(3)	4635(3)	3991(3)	34(1)
C(6)	4815(4)	3535(3)	4695(3)	33(1)
C(7)	4851(5)	6834(3)	4012(3)	50(1)
C(8)	6041(6)	6982(4)	3312(4)	69(1)

Table S7. Anisotropic displacement parameters ($Å^2 \times 10^3$) for EDABCO-$CuCl_4$. The anisotropic displacement factor exponent takes the form: $-2p^2[h^2a^{*2}U^{11} + ... + 2 h k a^* b^* U^{12}]$

	U^{11}	U^{22}	U^{33}	U^{23}	U^{13}	U^{12}
Cu(1)	34(1)	28(1)	31(1)	0(1)	-2(1)	2(1)
Cl(1)	66(1)	57(1)	29(1)	-2(1)	-6(1)	6(1)
Cl(2)	67(1)	26(1)	34(1)	-1(1)	-2(1)	9(1)
Cl(3)	28(1)	36(1)	56(1)	-4(1)	0(1)	1(1)
Cl(4)	31(1)	31(1)	46(1)	-1(1)	-5(1)	-1(1)
N(1)	37(2)	22(1)	25(1)	-1(1)	-4(1)	-1(1)
N(2)	31(1)	28(1)	31(1)	5(1)	-1(1)	-2(1)
C(1)	33(2)	26(2)	37(2)	-6(1)	-6(1)	-5(1)
C(2)	36(2)	34(2)	37(2)	1(2)	-11(1)	-5(2)
C(3)	35(2)	41(2)	35(2)	0(2)	1(2)	14(2)
C(4)	30(2)	49(2)	33(2)	1(2)	7(1)	1(2)
C(5)	43(2)	31(2)	28(2)	-6(1)	0(1)	-9(1)
C(6)	38(2)	27(2)	35(2)	-7(1)	4(1)	-6(2)
C(7)	81(3)	30(2)	38(2)	10(1)	-12(2)	-8(2)
C(8)	95(4)	52(2)	61(3)	4(2)	13(3)	-27(3)

References

1 Islam, R. A. & Priya, S. Realization of high-energy density polycrystalline piezoelectric ceramics. *Appl. Phys. Lett.* **88**, 032903 (2006).

2 Randall, C. A., Kim, N., Kucera, J. P., Cao, W. W. & Shrout, T. R. Intrinsic and extrinsic size effects in fine-grained morphotropic-phase-boundary lead zirconate titanate ceramics. *J. Am. Ceram. Soc.* **81**, 677–688 (1998).

3 Suwannasiri, T. S., A. Effect of Rare-Earth Additives on Electromechanical Properties of Modified Lead Titanate Ceramics. *J. Am. Ceram. Soc.* **76**, 3155 (1993).

4 Ikegami, S., Ueda, I. & Nagata, T. Electromechanical Properties of $PbTiO_3$ Ceramics Containing La and Mn. *J. Acoust. Soc. Am.* **50**, 1060–1066 (1971).

5 Liu, B. *et al.* Simultaneously enhanced piezoelectric response and piezoelectric voltage coefficient in textured KNN-based ceramics. *J. Am. Ceram. Soc.* **101**, 265–273 (2018).

6 Yang, Z., Zhou, S., Zu, J. & Inman, D. High-Performance Piezoelectric Energy Harvesters and Their Applications. *Joule* **2**, 642-697 (2018).

7 Yan, Y., Zhou, J. E., Maurya, D., Wang, Y. U. & Priya, S. Giant piezoelectric voltage coefficient in grain-oriented modified $PbTiO_3$ material. *Nat. Commun.* **7**, 13089 (2016).

8 You, Y. M. *et al.* An organic-inorganic perovskite ferroelectric with large piezoelectric response. *Science* **357**, 306–309 (2017).

9 Zhang, Y. *et al.* A molecular ferroelectric thin film of imidazolium perchlorate that shows superior electromechanical coupling. *Angew Chem Int. Ed* **53**, 5064–5068 (2014).

10 Shepelin, N. A. *et al.* New developments in composites, copolymer technologies and processing techniques for flexible fluoropolymer piezoelectric generators for efficient energy harvesting. *Energy Environ. Sci.* **12**, 1143–1176 (2019).

11 Fu, D. W. *et al*. Diisopropylammonium bromide is a high-temperature molecular ferroelectric crystal. *Science* **339**, 425 (2013).

12 Chen, X. G. *et al.* Two-Dimensional Layered Perovskite Ferroelectric with Giant Piezoelectric Voltage Coefficient. *J. Am. Chem. Soc.* **142**, 1077–1082 (2020).

13 Hu, Y. *et al.* Bond engineering of molecular ferroelectrics renders soft and high-performance piezoelectric energy harvesting materials. *Nat. Commun.* **13**, 5607 (2022).

14 Huang, G. *et al.* Achieving Ultrahigh Piezoelectricity in Organic–Inorganic Vacancy-Ordered Halide Double Perovskites for Mechanical Energy Harvesting. *ACS Energy Lett.* **6**, 16–23 (2020).

15 Deswal, S. *et al.* Flexible Composite Energy Harvesters from Ferroelectric A_2MX_4-Type Hybrid Halogenometallates. *Chem. Mater.* **31**, 4545–4552 (2019).

16 Ding, R. *et al.* High-performance piezoelectric nanogenerators composed of formamidinium lead halide perovskite nanoparticles and poly(vinylidene fluoride). *Nano Energy* **37**, 126–135 (2017).

17 Khan, A. A. *et al.* Superior transverse piezoelectricity in organic-inorganic hybrid perovskite nanorods for mechanical energy harvesting. *Nano Energy* **86**, 106039 (2021).

18 Sahoo, S. *et al.* Ferroelectricity and Piezoelectric Energy Harvesting of Hybrid A_2BX_4-Type Halogenocuprates Stabilized by Phosphonium Cations. *ACS Mater. Au* **2**, 124–131 (2021).

19 Pandey, R. *et al.* Microscopic Origin of Piezoelectricity in Lead-Free Halide Perovskite: Application in Nanogenerator Design. *ACS Energy Lett.* **4**, 1004–1011 (2019).

20 Ippili, S., Jella, V., Kim, J., Hong, S. & Yoon, S. G. Unveiling Predominant Air-Stable Organotin Bromide Perovskite toward Mechanical Energy Harvesting. *ACS Appl. Mater. Interfaces* **12**, 16469–16480 (2020).

21 Khan, A. A. *et al.* Maximizing piezoelectricity by self-assembled highly porous perovskite–polymer composite films to enable the internet of things. *J. Mater. Chem. A* **8**, 13619–13629 (2020).

22 Sultana, A. *et al.* Methylammonium Lead Iodide Incorporated Poly(vinylidene fluoride) Nanofibers for Flexible Piezoelectric-Pyroelectric Nanogenerator. *ACS Appl. Mater. Interfaces* **11**, 27279–27287 (2019).

23 Rana, M. M. *et al.* Enhanced piezoelectricity in lead-free halide perovskite nanocomposite for self-powered wireless electronics. *Nano Energy* **101**, 107631 (2022).

24 Ippili, S. *et al.* An eco-friendly flexible piezoelectric energy harvester that delivers high output performance is based on lead-free $MASnI_3$ films and $MASnI_3$-PVDF composite films. *Nano Energy* **57**, 911–923 (2019).

25 Vijayakanth, T., Ram, F., Praveenkumar, B., Shanmuganathan, K. & Boomishankar, R. Piezoelectric Energy Harvesting from a Ferroelectric Hybrid Salt $[Ph_3MeP]_4[Ni(NCS)_6]$ Embedded in a Polymer Matrix. *Angew Chem Int. Ed* **59**, 10368–10373 (2020).
